# The association between anemia and sensorineural hearing loss: A review

**DOI:** 10.1097/MD.0000000000040326

**Published:** 2024-11-01

**Authors:** Liting Ye, Dong Lai, Junhu Tai

**Affiliations:** a Department of Transfusion, The Second Affiliated Hospital of Xiamen Medical College, Xiamen, China; b Department of Otorhinolaryngology-Head & Neck Surgery, The Second Affiliated Hospital of Xiamen Medical College, Xiamen, China.

**Keywords:** anemia, hemoglobin, hereditary disorder, nutrition, sensorineural hearing loss

## Abstract

Anemia affects a third of the world’s population and contributes to increased morbidity and mortality, decreased work productivity, and impaired neurological development. In recent years, many studies have found a possible association between anemia and sensorineural hearing loss (SNHL), especially in various types of nutritional deficiency and hemoglobin disorders anemia. Anemia may affect hearing through various mechanisms, including affecting microcirculation in the ear, causing tissue hypoxia in the ear, and through inflammatory and oxidative stress pathways. This review aims to comprehensively analyze the association between various types of anemia and SNHL, including possible biological mechanisms, clinical features, and treatment strategies, and clarify the importance of anemia treatment and management in preventing SNHL.

## 1. Introduction

Anemia is a global disease that affects the health of the population, and according to statistics, 1 in every 3 people suffers from anemia.^[1]^ Anemia can be classified into different types based on red blood cell size, severity, and cause of onset, such as iron-deficiency anemia (IDA), aplastic anemia, thalassemia, and so on.^[2]^ Among them, IDA accounts for half of all types of anemia.^[3]^ IDA can lead to neurodevelopmental defects, and even after correcting IDA, this defect may not always be fully restored.^[4]^ Sensorineural hearing loss (SNHL) is a type of hearing loss that typically leads to difficulties in understanding speech and other sounds, seriously affecting the work and life of the population.^[5]^ SNHL accounting for approximately 90% of all hearing loss, and the main causes of SNHL include advanced age, ototoxic drugs, noise exposure, and genetic and autoimmune diseases.^[6]^ Moreover, anemia is also considered one of the important causes of SNHL.

More and more evidence suggests that anemia is associated with SNHL, but the exact mechanism of anemia leading to SNHL is still unclear. However, it has been widely hypothesized to be related to the sensitivity of the cochlea to vascular and neurologic.^[7]^ Due to the fact that the cochlea is supplied only by the labyrinthine artery, the lack of collateral circulation could make it more vulnerable to the ischemic.^[8]^ A meta-analysis aimed to investigate the relationship between anemia and SNHL, involving over 300,000 individuals.^[9]^ The results showed that individuals with anemia had a more than 50% higher risk of developing SNHL compared to those without anemia. Another study evaluated the relationship between the severity of anemia and hearing loss, and the results showed that over 40% of moderate anemic patients and over 60% of severe anemic patients had SNHL.^[10]^ There is a significant correlation between the degree of anemia and SNHL. This article aims to review the relationship between various types of anemia and SNHL, report relevant clinical and basic research results, analyze the correlation and possible biological mechanisms of the 2 diseases, and introduce existing treatment strategies and intervention measures, providing some information and tips for future research and treatment.

This study searched PubMed, Web of Science, and Scopus, using a combination of the following search terms (in title/abstract): “anemia” and “sensorineural hearing loss.” There are no restrictions on the types of articles. Article types such as review, original research, letters, communications, and editorials are all included in this review, only some articles that could not find the full text were excluded. All selected articles have been imported into EndNote, which will intelligently delete duplicate articles.

## 2. IDA and SNHL

IDA, as the most prevalent subtype among different types of anemia, patients exhibit low hemoglobin, serum ferritin, serum iron, and increased soluble transferrin receptor. IDA is usually caused by blood loss or insufficient iron absorption, and has a good response to relieving the cause of blood loss and oral iron supplementation.^[11]^ The metabolism of neurotransmitters, DNA synthesis, and DNA repair mechanisms all rely on iron as a cofactor, and iron is also a key component of hemoglobin, which is a protein in red blood cells responsible for transporting oxygen to body tissues.^[12]^ The important role of iron in vasculature and nervous system explains the possibility that iron deficiency will lead to SNHL. A comparative study reported the relationship between IDA and SNHL. According to their speculation, the blood supply to the inner ear, which is highly susceptible to ischemic injury, is damaged by IDA, leading to SNHL.^[13]^ A large retrospective cohort study in the United States showed a significant correlation between SNHL and IDA, especially in the population under 60 years old. But this study is only targeted at adults seeking healthcare, and the results may not be generalizable to other populations.^[14]^ The experimental results of another study on the effects of iron deficiency on cochlear development in rats showed that the main cochlear histopathological changes induced by iron deficiency were strial atrophy and reduction of spiral ganglion cells, and the abnormalities may be entirely attributed to iron deficiency in cochlear tissue.^[15]^ There is a report introducing a case of SNHL related to IDA.^[16]^ A 49-year-old female received intravenous hydrocortisone treatment for SNHL, but her hearing loss did not recover. Subsequently, after intravenous administration of sucrose iron for IDA, the patient’s hearing loss gradually recovered within 2 months. Given the elevated serum hemoglobin levels after iron treatment, it suggests that the patient’s SNHL may be related to the IDA. In a randomized study involving over 400 patients with SNHL, patients receiving iron therapy showed significantly better efficacy than those receiving other treatment options, and more than half of the patients’ hearing has improved.^[17]^ Iron therapy is an effective treatment for IDA-related SNHL, and the serum hemoglobin and iron levels of such patients with SNHL should be checked to provide better treatment.

## 3. Megaloblastic anemia and SNHL

There are many causes of megaloblastic anemia, and the most common reason is a deficiency of vitamin B_12_ or folate. The diagnostic process typically involves first determining the presence of B_12_ or folate deficiency, and then identifying the cause of the deficiency.^[18]^ Megaloblastic anemia contains various clinical features, mainly including anemia, cytopenias, jaundice, and megaloblastic marrow morphology.^[19]^ In megaloblastic anemia, a rare autosomal recessive disorder is associated with SNHL. Thiamine-responsive megaloblastic anemia syndrome (TRMA), is an autosomal recessive disease caused by loss-of-function mutations in the SLC19A2 gene, which encodes the human thiamine transporter 1 protein, the main transporter of thiamine in many tissues.^[20]^ The disruption of the thiamine transport mechanism will lead to apoptosis and functional impairment of cochlear cells, pancreatic islet cells, and hematopoietic stem cells.^[21]^ TRMA has triad symptoms, including megaloblastic anemia, SNHL, and non-type 1 diabetes mellitus.^[22]^ Among them, non-type 1 diabetes mellitus and megaloblastic anemia are responsive to high-dose thiamine treatment, and active treatment can delay or prevent their onset.^[23]^ However, the symptoms of SNHL are progressive and irreversible, and will not be affected or prevented by early puberty treatment. This may be because cochlear cells are highly sensitive to thiamine deficiency, and hearing loss may manifest as early as fetal development.^[24]^ A study from Italy involved 4 patients with TRMA who received high-dose thiamine treatment, but their SNHL did not improve.^[25]^ Current research suggests that hearing aids also have little effect, and only cochlear implantation seems to solve SNHL caused by TRMA.^[26]^ But there is also a study that suggests that using thiamine before 2 months after birth may effectively prevent hearing loss.^[27]^ These studies suggest that thiamine treatment in the early stages of cochlear development is one of the methods to address SNHL caused by TRMA.

## 4. Aplastic anemia and SNHL

Aplastic anemia is a rare hematopoietic stem cell disease that can lead to pancytopenia and hypocellular bone marrow. The pathophysiology of aplastic anemia is immune-mediated, with self-reactive lymphocytes mediating the destruction of hematopoietic stem cells. Factors such as drugs or viruses are believed to trigger abnormal immune responses in patients with aplastic anemia.^[28]^ There are few research reports on the correlation between aplastic anemia and SNHL. A study in Japan reported 3 cases of patients with aplastic anemia developing SNHL, who suddenly experienced severe hearing loss, and none of the 3 patients’ hearing was restored after active treatment. The author suggests that cochlear hemorrhage may be one of the reasons for SNHL in these patients.^[29]^ Another study in Taiwan involved 14 patients with aplastic anemia, whose percentages of recruitment phenomenon showed a significant difference between endolymphatic hydrops and SNHL. This suggests that SNHL in patients with aplastic anemia may be related to endolymphatic hydrops.^[30]^ Fanconi anemia is a rare genetic hematological disorder characterized by typical symptoms of aplastic anemia such as bleeding tendency.^[31]^ Hearing loss is also one of the many clinical manifestations of this disease. Although the most common audiological manifestation of Fanconi anemia is asymmetrical bilateral conductive hearing loss,^[32]^ SNHL also occurs in Fanconi anemia. A study conducted in the Netherlands showed that among 29 patients with Fanconi anemia, 16 showed hearing loss, including 6 cases of SNHL, 7 cases of conductive hearing loss, and 3 cases of mixed hearing loss.^[33]^ Additionally, they collected data on speech perception in noise difficulties. And found that speech perception in noise was subnormal in over half of the patients with Fanconi anemia. In terms of treatment, besides active treatment for the primary disease and hearing aids, there are no reports of additional special treatments, and further research is needed.

## 5. Sickle cell anemia and SNHL

Sickle cell anemia (SCA) is a hereditary disorder of hemoglobin, and the sickle mutation is permanent, causing great distress to patients.^[34]^ The clinical manifestations of SCA mainly include vaso-occlusive crises, hemolytic crises, chronic anemia, pain, stroke, infections, avascular necrosis, and so on.^[35]^ Considering the vaso-occlusive nature of SCA, there is a high possibility of hearing loss. Known studies have shown that SCA has auditory pathway dysfunction. Its physiological and pathological mechanisms are related to the circulatory changes of the auditory system, which may be caused by abnormal shapes of blood cells, leading to structural hypoxia of the auditory system and resulting in hearing loss.^[36]^ Various studies suggest that the incidence of SNHL is higher in patients with SCA than in healthy subjects.^[37]^ According to reports, the incidence of SNHL is increasing in children with SCA.^[38]^ Among 181 children with SCA, 24 suffered from hearing loss, of which half were SNHL. Their high-frequency hearing loss is more severe than their low-frequency hearing loss. Another study showed that 15 out of 52 children with SCA developed SNHL, which was associated with endothelial dysfunction, suggesting that inflammation in SCA can reduce blood flow to the inner ear by damaging endothelial cells. Therefore, they considered that this circulatory disorder leads to vaso-occlusive and induces auditory disorders.^[39]^ A meta-analysis aimed to evaluate the prevalence of SNHL in the adult SCA population, including 12 studies involving 636 patients and 360 controls. The results showed that SNHL is more common in SCA patients, especially those with Hb SS genotype, and they explained that this may be due to the impact of SCA on the labyrinthine microvasculature.^[40]^ Another meta-analysis included 21 studies, and the results showed that the prevalence of SNHL in SCA was 20.5%, most likely due to the presence of circulatory disorders caused by the chronic inflammatory state of SCA.^[41]^ In terms of treatment, early intervention is more important. However, after the development of severe SNHL, the effectiveness of hearing aids is unsatisfactory, and cochlear implantation is needed to address SNHL caused by SCA.^[42]^ Patients with SCA face issues including psychological and economic pressure, workplace discrimination, and so on. Early identification and intervention, enhanced pain management strategies, and mental health care are crucial for reducing social and psychological distress and improving the lives of patients with SCA.^[43]^

## 6. Thalassemia and SNHL

Thalassemia is one of the common genetic diseases, in which affected individuals have variable expression of alpha or beta chains, leading to an imbalance in their utilization in processes such as hemoglobin formation.^[44]^ Thalassemia is classified into α- and β-thalassemia based on which of the globin chains is affected.^[45]^ Thalassemia can cause significant red blood cell damage and severe hemolysis, and in β-thalassemia, it can also cause deformation of the skull and face. In addition, thalassemia can cause extensive lymphadenopathy and hepatosplenomegaly.^[46]^ In addition to these symptoms, many studies have reported cases of SNHL caused by β-thalassemia. A multicenter case-control study in Italy showed that the incidence rate of SNHL in β-thalassemia reached more than 20%, and the incidence rate in men was higher than that in women.^[47]^ Another study involving over 100 patients with β-thalassemia also showed that about 20% of patients developed SNHL, as these patients all received deferoxamine (DFO) chelation treatment, suggesting that DFO plays an important role in the development of SNHL.^[48]^ A meta-analysis involving 17 articles and over 1800 patients with thalassemia receiving DFO treatment showed that the prevalence of SNHL reached 10.6%, and hearing loss was not related to the average daily amount of DFO treatment, but to the duration of treatment.^[49]^ In terms of evaluating whether long-term iron chelation therapy leads to SNHL in patients with β-thalassemia, a longitudinal follow-up study over 20 years reported that the incidence of SNHL in patients with β-thalassemia receiving iron chelation therapy with DFO, deferiprone, or deferasirox was 23.8%, and hearing loss was significantly associated with chelation duration.^[50]^ These results suggest that iron chelation therapy in the treatment of β-thalassemia may induce ototoxicity and increase the probability of SNHL in patients with β-thalassemia. It is necessary to conduct long-term and accurate hearing follow-up for them.

## 7. Conclusions

Numerous clinical reports and basic research indicate that various types of anemia are more or less related to SNHL. For example, patients with IDA may develop SNHL due to iron deficiency, patients with TRMA suffer from SNHL due to disruption of the thiamine transport mechanism, patients with aplastic anemia may suffer from SNHL due to cochlear hemorrhage or endolymphatic hydrops, patients with SCA may suffer from SNHL due to impaired labyrinthine microvasculature, and patients with thalassemia may suffer from SNHL due to ototoxicity caused by iron chelation therapy (Table 1). The specific pathophysiological mechanisms and specific treatment methods for SNHL caused by these reasons still need further exploration and research.

**Table 1:** Summary of different types of anemia and SNHL.

Type of anemia	Possible mechanisms of hearing loss
Iron-deficiency anemia	Iron deficiency affects the blood supply to the inner ear
Megaloblastic anemia (thiamine-responsive megaloblastic anemia)	Disruption of the thiamine transport mechanism leads to apoptosis and functional impairment of cochlear cells
Aplastic anemia	Cochlear hemorrhage, endolymphatic hydrops
Sickle cell anemia	Impaired labyrinthine microvasculature
Thalassemia	Iron chelation therapy induce ototoxicity

SNHL = sensorineural hearing loss.

## Author contributions

**Writing—original draft:** Liting Ye, Dong Lai.

**Writing—review & editing:** Junhu Tai, Dong Lai.

**Conceptualization:** Junhu Tai.
